# Carbon dioxide Angiography-Guided Renal-Related Interventions in Patients with Takayasu Arteritis and Renal Insufficiency

**DOI:** 10.1007/s00270-018-1936-x

**Published:** 2018-03-16

**Authors:** Sujith Chacko, George Joseph, Viji Thomson, Paul George, Oommen George, Debashish Danda

**Affiliations:** 1 Department of Cardiology, Christian Medical College, Vellore, 632004 India; 2 Department of Rheumatology, Christian Medical College, Vellore, India

**Keywords:** Renal insufficiency, Renal failure, Takayasu arteritis, Carbon dioxide, Angioplasty, Stent, Renal artery stenosis, Aortic stenosis, Dissection, Pseudoaneurysm

## Abstract

**Background:**

Use of iodinated contrast agents for angiography in patients with renal insufficiency risks further deterioration of renal function and its adverse sequelae.

**Objective:**

To study the effectiveness and safety of carbon dioxide (CO_2_) angiography in guiding percutaneous renal-related interventions in patients with Takayasu arteritis and renal insufficiency.

**Methods:**

Data on CO_2_ angiography-guided interventions were obtained from a 23-year database of 692 Takayasu arteritis patients who underwent percutaneous interventions and were analyzed retrospectively. Follow-up data were also obtained. The CO_2_ angiography system used was developed in-house and was pressure-driven.

**Results:**

Seven patients (6 female, age 16–59 years, baseline serum creatinine 1.62–4.55 mg/dl, estimated glomerular filtration rate 12.2–36.9 ml/min/1.73 m^2^) underwent CO_2_ angiography-guided interventions: five underwent angioplasty or stenting to treat six stenotic/occluded renal arteries, one underwent extensive endovascular repair for spontaneous focal abdominal aortic dissection with false lumen aneurysm and aorto-iliac true lumen narrowing, and one underwent balloon dilatation of previously deployed aortic stents used to treat aortic occlusion at two levels. Follow-up (median 5 years, range 2 months–16 years) was obtained in all patients. All the procedures were successful and resulted in relief of symptoms, better blood pressure control, improvement in left ventricular systolic function and recovery or stabilization of renal function. There were no early or late complications related to CO_2_ angiography. Three renal lesions that had restenosis at follow-up were managed successfully by repeat intervention.

**Conclusion:**

CO_2_ angiography-guided renal-related interventions are effective and safe in patients with Takayasu arteritis and renal insufficiency; they significantly improve the care of such patients.

## Introduction

Carbon dioxide (CO_2_) has been used as an intra-arterial contrast agent during angiography for more than four decades [1]. Renal insufficiency is an important indication for the use of CO_2_ angiography, given the absence of nephrotoxicity with this agent [2]. Takayasu arteritis (TA), a chronic idiopathic granulomatous large vessel vasculitis affecting the aorta and its main branches [3], can produce renal insufficiency [4], mainly by causing renal artery stenosis [5]. At our center, prevalence of renal dysfunction in 118 patients with TA presenting during the period 1963–1977 was 13.6% [6]. A recent study by Li et al. [4] showed an overall prevalence of renal dysfunction of 11.4% in 411 patients with TA. There are no reports of the use of CO_2_ angiography-guided interventions in TA apart from a single case report from our center in 2003 [7]; the present study describes our 16-year single-center experience with CO_2_ angiography in guiding renal-related percutaneous interventions in patients with TA.

## Methods

### Patients

Data on percutaneous interventions performed on patients with TA at our center (a large tertiary care referral hospital in South India) that were collected prospectively over 23 years and archived digitally were scrutinized. Cases where CO_2_ angiography was utilized to guide percutaneous interventions were selected, and the relevant case records and angiographic images were analyzed. Follow-up was obtained in all patients at 6- to 12-month intervals if stable, but more frequently when indicated. Clinical status, imaging information and additional procedures done at follow-up were studied. All procedures were performed after obtaining written informed consent. The institutional review board approved this retrospective study. For this type of study formal consent is not required.

### Carbon dioxide Angiography System

**Fig. 1:**
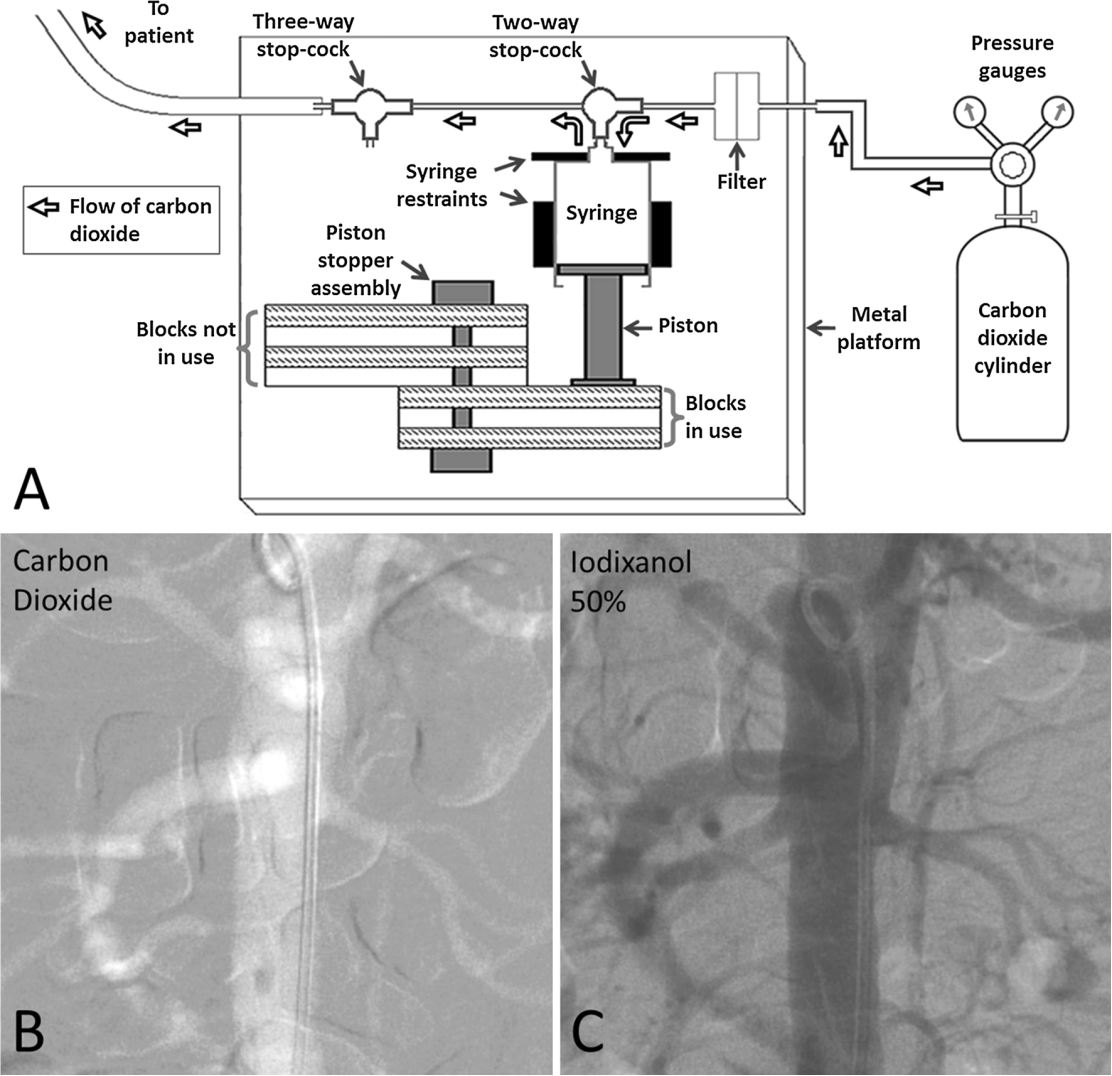
**A** Diagrammatic representation of the apparatus used for carbon dioxide angiography. Every alternate block of the piston stopper assembly has been left un-shaded to reveal the perpendicular rod around which the blocks can swivel 180°; all the blocks are identical. **B, C.** Consecutive abdominal aortograms performed in antero-posterior projection in a patient with renal failure using carbon dioxide and 50% iodixanol, respectively, with identical settings of digital subtraction angiography

The CO_2_ angiography system used (Fig. 1A) was developed in-house and has been operational for the last 17 years. It has no moving parts, and CO_2_ injection is pressure-driven. Pressurized medical grade CO_2_ obtained from a storage cylinder is passed through a Millipore filter into a disposable 50 ml plastic collection syringe that is held firmly between fixed restraints on a metal platform. The piston of the collection syringe is variably restrained by an assembly of swivelling blocks; the volume of the syringe is determined by the number of blocks in use in the piston stopper assembly. A two-way stopcock attached to the collection syringe nozzle ensures that at no stage can the flow of CO_2_ bypass the syringe and go directly from cylinder to patient. Gas pressure in the collection syringe is kept at 4 kg/cm^2^ (nearly 4 atm) by appropriately adjusting the knob in the dual pressure gauge assembly which separately indicates cylinder (inflow) and tubing (outflow) pressures. At this setting, the volume of CO_2_ delivered into the vessel is approximately three times the selected volume in the collection syringe; this can be verified by trial injection of CO_2_ into a collapsed plastic bag attached to the three-way stopcock along the tubing. Prior to angiography, the tubing and angiographic catheter are flushed with CO_2_ to remove air and avoid explosive delivery of CO_2_. For CO_2_ injection, the two-way stopcock is turned 90° to allow CO_2_ to flow from the collection syringe to the patient. Standard protocols were used for patient preparation and digital subtraction angiography. Typically, the collection syringe volume was set at 20 ml for flush aortograms (to deliver 60 ml of CO_2_) and 10 ml for selective angiograms (to deliver 30 ml of CO_2_). The quality of images obtained using this CO_2_ angiography system compared favorably with that obtained with 50% iodixanol, a diluted alternative contrast agent, that is sometimes used in renal insufficiency (Fig. 1B, C).

## Results

Of 692 patients with TA who underwent percutaneous interventions to treat 1834 diseased arteries (including 425 renal arteries) over a 23-year period, seven patients (1.01%) underwent CO_2_ angiography-guided percutaneous interventions because of renal insufficiency (Table 1). All seven patients (1 male/6 female, mean age 38 years) met both the American College of Rheumatology [8] and the modified Ishikawa (clinical) criteria [9] for the diagnosis of TA; all were hypertensive on multiple medications. All patients were symptomatic at presentation, often with recent worsening.

**Table 1:** Case and procedure details

Feature		Case 1	Case 2	Case 3	Case 4	Case 5	Case 6	Case 7
*Baseline parameters*								
Age (years)/Sex		59 F	45 M	30 F	28 F	38 F	50 F	16 F
Takayasu arteritis diagnostic criteria met								
ACR criteria		5 of 6	5 of 6	5 of 6	3 of 6	5 of 6	3 of 6	4 of 6
Clinical criteria		2 major, 4 minor	2 major, 3 minor	2 major, 4 minor	1 major, 2 minor	1 major, 3 minor	1 major, 4 minor	1 major, 5 minor
Limb blood pressure (mmHg)								
Upper		180/100	210/130	220/110	210/130	114/64	179/83†	160/96
Lower		220/120	170/100	220/110	n/a	139/69	76/37†	65 mean†
Number of anti-HTN drugs		4	3	6	3	5	4	2
LV ejection fraction (%)		56	56	35	n/a	40	37	38
Creatinine (mg/dl)		3.27	3.50	1.62	1.80	4.55	3.39	1.90
eGFR (ml/min/1.73 m^2^)		17.0	21.3	36.9	32.8	12.2	17.9	33.1
Kidney size (cm)								
Right		9.0	Removed	9.8	11.8	9.7	7.0	8.8
Left		6.9	10.4	9.2	Removed	Shrunken	8.6	Shrunken
Renal artery status								
Right		70% proximal stenosis	Absent (post-nephrectomy)	Ostial occlusion	90% ostial stenosis	90% distal edge stenosis	Occluded	Occluded
Left		Occluded	90% stenosis proximally	80% ostial stenosis	Absent (post- nephrectomy)	Occluded	Normal	Occluded
Abdominal aorta status		Mildly ectatic aorta, irregular in outline	Long infra-renal aortic stenosis with 36 mmHg peak gradient	Minor infra-renal narrowing	Minor infra-renal narrowing	Patent stent in infra-renal aorta	Focal dissection with TL narrowing and FL aneurysm; occluded SMA IMA	Aortic occlusions above the celiac and below the renal arteries
Presentation		Uncontrolled HTN, blurring of vision, worsening renal function	Uncontrolled HTN, fatigue, pedal edema, worsening renal function	Acute pulmonary edema, absent left upper limb pulses, renal bruit, HTN	Uncontrolled HTN, pedal edema	Recent pulmonary edema with accelerated HTN – now controlled	Abdominal pain, bilateral lower limb claudication	Dyspnea on exertion, bilateral lower limb claudication
*Interventional procedures*								
Carbon dioxide angiography-guided procedures		Right renal stenting	Left renal stenting	Bilateral renal stenting	Right renal stenting	Balloon angioplasty of right renal stent	Aortic endograft, left renal + celiac chimney stents, aorto-iliac stents	Dilatation of aortic stents
Volume of iodinated contrast used with above		Nil	Nil	Nil	Nil	Nil	4 ml	Nil
Earlier or later renal or aortic procedures not requiring carbon dioxide angiography		None	Right nephrectomy 4 years earlier. Infra-renal aortic stenting 6 years later	Angioplasty for bilateral renal in-stent restenosis 5 years later	Left nephrectomy 14 years earlier. Angioplasty for right renal in-stent restenosis 3 and 6 years later	Both renal arteries, descending and abdominal aorta stented 1-16 years earlier. Left renal stent occluded.	None	Two-segment aortic stenting 7 months earlier. Right renal auto-transplantation 2 weeks later
*Follow-up status*								
Duration of follow-up		15 months	13 years	5 years	12 years	2 months	12 months	16 years
Limb blood pressure (mmHg)								
Upper		130/90	110/70	150/80	110/70	114/72	160/50	126/70
Lower		n/a	96 systolic	150 systolic	120/80	140 systolic	170 systolic	136 systolic
Number of anti-HTN drugs		1	2	2	2	3	5	2
LV ejection fraction (%)		n/a	55	69	56	n/a	49	58
Creatinine (mg/dl)		1.20	1.35	0.79	0.86	0.98	3.52	0.84
eGFR (ml/min/1.73m2)		39.5	55.1	75.7	72.5	56.4	17.2	75.0
Symptoms		Asymptomatic	Asymptomatic	Asymptomatic	Asymptomatic	Asymptomatic	Asymptomatic	Asymptomatic
Angiographic/Doppler findings		Awaited	Left renal and aortic stents widely patent	Awaited after angioplasty for bilateral restenosis	Normal right renal flow pattern	Awaited	Patent stents with normal flow pattern	Widely patent aortic stents and transplant renal artery

*M* male, *F* female, *ACR* American College of Rheumatology, *HTN* hypertension, *LV* left ventricle, *n/a* data not available, *eGFR* estimated glomerular filtration rate, *ESR* erythrocyte sedimentation rate, *TL* true lumen, *FL* false lumen, *SMA* superior mesenteric artery, *IMA* inferior mesenteric artery

^¶^Some aspects of case 4 have been published earlier Ref. [7]

†Intra-arterial pressure

*Angiographic appearance

### Renal Interventions

**Fig. 2:**
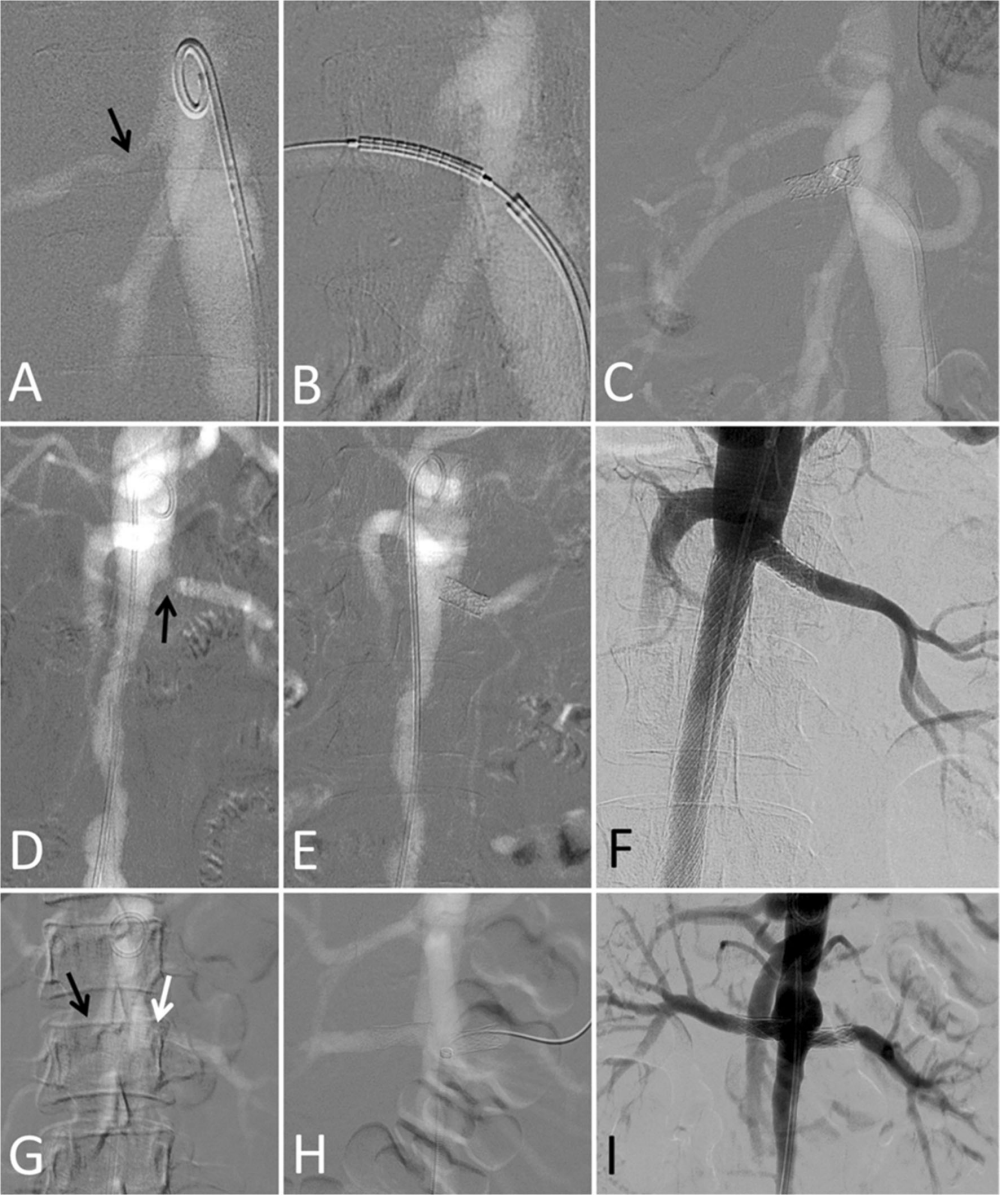
Carbon dioxide-guided renal artery interventions in patients with Takayasu arteritis and renal insufficiency. All images are carbon dioxide angiograms unless stated otherwise. **A to C.** Case 1. Baseline angiogram (**A**) shows right renal artery stenosis (arrow). After stent positioning (**B**) and deployment, the final angiogram (**C**) showed a good outcome. **D to F.** Case 2. Baseline angiogram (**D**) shows ostial left renal artery stenosis (arrow) and long infra-renal aorta narrowing. Renal function normalized after left renal stenting (**E**). Conventional angiogram (**F**) obtained 11 years later (5 years after interval infra-renal aortic stenting) shows good long-term outcome. **G to I.** Case 3. Baseline angiogram (**G**) shows right renal artery occlusion (black arrow) and left renal artery stenosis (white arrow). Bilateral renal artery stenting was performed (**H**) leading to normalization of renal function. Conventional angiogram done 4 months later (**I**) shows good short-term outcome

Five patients with significant renal artery stenosis underwent CO_2_ angiography-guided renal angioplasty or stenting using percutaneous femoral arterial access (Table 1, cases 1–5; Fig. 2). In cases 1, 2 and 4, the renal artery was engaged with a 7F renal-guiding catheter and the stenotic lesion was crossed with a 0.018-inch nitinol guidewire. In cases 3 and 5, the renal arteries were engaged with a 6F Judkins Right diagnostic catheter and a 0.035-inch angled hydrophilic guidewire was used to cross the renal artery lesion; this was replaced with a 0.035-inch stiff guidewire with 1-cm soft tip over which a long 7F femoral sheath was advanced up to the renal artery ostium. Balloon-expandable bare metal stents were deployed in all the lesions except in case 5 where a stent had been deployed earlier. Balloon-dilatation pressures of 8 to 14 atm were used. A satisfactory result was obtained in all vessels without complications.

### Aortic Interventions

**Fig. 3:**
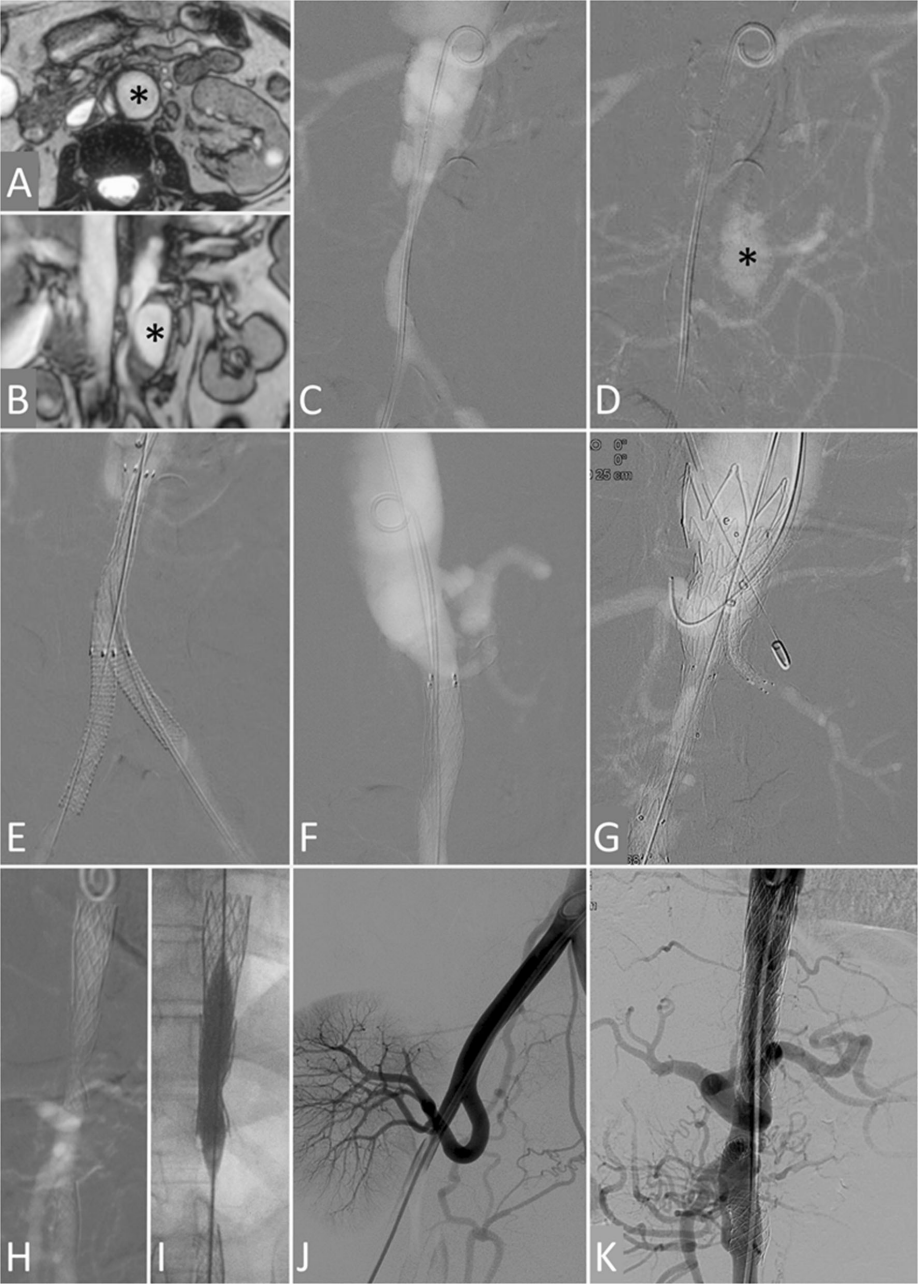
Carbon dioxide-guided aortic and ancillary interventions in patients with Takayasu arteritis and renal insufficiency. **A to G.** Case 6. Magnetic resonance angiograms in transverse (**A**) and coronal (**B**) planes and carbon dioxide angiogram early (**C**) and late (**D**) frames in antero-posterior (AP) projection show a single (left) renal artery, focal abdominal aortic dissection, narrowing of the infra-renal aorta and both common iliac arteries and a large false lumen aneurysm on the left lateral aspect of infra-renal aorta (black asterisk). Carbon dioxide AP (**E**) and lateral (**F**) angiograms after infra-renal aortic and bilateral iliac artery stenting show relief of stenosis; the superior mesenteric and right renal arteries are not visualized. Carbon dioxide AP angiogram (**G**) after deployment of a tapered endograft in the abdominal aorta and chimney grafts in the left renal and superior mesenteric arteries shows patency of these arteries; the false lumen aneurysm was no longer visualized in the late frames. **H to K.** Case 7. Carbon dioxide AP angiogram (**H**) obtained 7 months after stenting of the lower thoracic-upper abdominal and infra-renal aorta (renal function had deteriorated since then) shows residual stenosis and non-visualization of the renal arteries. The stents were further expanded by balloon dilatation (**I**), and the right kidney was auto-transplanted (**J**) resulting is normalization of renal function. Conventional AP aortogram (**K**) obtained 11 years later shows widely patent aortic stents

Two patients underwent CO_2_ angiography-guided aortic and ancillary interventions with successful outcomes and without complications (Table 1, cases 6, 7; Fig. 3).

In case 6, extensive endovascular repair was performed for spontaneous focal abdominal aortic dissection with false lumen aneurysm formation and aorto-iliac true lumen narrowing with 103 mmHg systolic pressure gradient. The celiac and left renal arteries were the only patent visceral aortic branches. In the first sitting, aorto-iliac stenting was performed with a covered stent in the infra-renal aorta and bare metal stents in both common iliac arteries. In a second sitting 7 days later, a tapered aorto-uni-iliac endograft was deployed in the abdominal aorta starting above the upper limit of the aortic dissection (at the celiac artery ostium level) and overlapping the earlier deployed stent below. Flow into the left renal artery was preserved by constructing a chimney graft. Flow into the celiac artery was preserved using a bare metal stent deployed in chimney fashion. A satisfactory result was obtained with exclusion of the false lumen aneurysm, abolition of the aorto-iliac pressure gradient and preserved flow into the left renal and celiac arteries.

Case 7 had presented 7 months earlier with occlusions of both renal arteries and of the aorta at two levels; the aortic occlusions were recanalized and stented with balloon-expandable stents, but the lesions were resistant to dilatation, and only partial opening was achieved with a 9-mm-diameter non-compliant balloon. Renal function had subsequently deteriorated, and in the present sitting CO_2_ angiography-guided balloon dilatation of both stents was performed using a 10-mm-diameter non-compliant balloon which resulted in further stent expansion and reduction in the aortic pressure gradient. This provided a sufficient pressure head for the right kidney to be auto-transplanted 2 weeks later to the right iliac fossa with the right renal artery being anastomosed end-to-end to the right internal iliac artery.

### Outcome and Follow-Up

CO_2_ angiography provided sufficiently clear visualization of the vascular anatomy and enabled achievement of successful outcomes in all the patients. Some patients felt mild transient abdominal discomfort immediately after CO_2_ injection, but none had nausea, vomiting, hypotension, narcosis or air contamination-related problems. There were no late complications related to CO_2_ angiography. All patients were followed-up after the CO_2_ angiography-guided intervention (median duration 5 years, range 2 months to 16 years; Table 1). None of the patients had deterioration in renal function or required dialysis; rather, renal function improved in 6 patients and stabilized in one. All patients experienced resolution of their symptoms. Blood pressure control improved, as also left ventricular systolic function that was initially depressed in some patients. Restenotic renal artery lesions seen at follow-up in cases 3 and 4 were treated by balloon dilatation; in case 3 this produced distal edge dissection in the right renal artery and required deployment of a stent. In case 7, progressive dilatation of the aortic stents was performed during subsequent follow-up visits.

### Comparison with Iodinated Contrast

During the same period of time, 46 TA patients with elevated serum creatinine levels (> 1.4 mg/dl) underwent interventions using iodinated contrast after intravenous hydration; of these, none with serum creatinine levels below 3.0 mg/dl developed sustained worsening of renal function or required dialysis, but one of two patients with serum creatinine ≥ 3.0 mg/dl required long-term dialysis. On the other hand, none of the TA patients who underwent CO_2_ angiography-guided interventions, including four with serum creatinine ≥ 3.0 mg/dl, had worsening of renal function or required dialysis.

## Discussion

Contrast-induced nephropathy is one of the most common causes of hospital-acquired renal insufficiency and is associated with a mortality of 14%; pre-existing renal insufficiency poses the greatest risk for developing this condition [10]. Absence of nephrotoxicity is perhaps the biggest advantage CO_2_ provides as a contrast agent, though it has several other benefits including being non-allergic and inexpensive, having low viscosity, allowing use of unlimited total volumes and not being diluted by blood [11]. With modern technology such as high-resolution digital subtraction angiography, stacking software, tilting tables and reliable delivery systems, the quality of images obtained with CO_2_ angiography has improved considerably [2]. In many cases, such as the ones presented in this report, the entire percutaneous intervention can be performed without use of iodinated contrast. In case 6, a single angiogram using 4 ml of iodinated contrast diluted with saline was used to confirm adequate collateral flow from the celiac artery to the superior and inferior mesenteric artery territories; this was done before deploying aortic endografts across the ostia of these already occluded vessels because recanalization of these vessels would no longer be possible. An important prerequisite for successful CO_2_ angiography is gentle, controlled, non-explosive delivery of the gas using a closed and ideally non-pressurized system; this can be achieved by use a series of one-way valves with a flaccid reservoir or blood bag [11, 12]. The CO_2_ angiography system used in this study was a pressurized system, but with adequate attention to outflow pressure and syringe volume and by purging the system with CO_2_ gas before use, no significant problems have been encountered; the system has worked well in more than 100 patients over 17 years, is simple, inexpensive and easy to use and has guided aorto-iliac, lower extremity, venous and complex endovascular procedures.

Renal insufficiency is not uncommon in TA despite these patients being young and having few co-morbidities [4, 6]. Renal insufficiency in TA is usually attributed to renal ischemia caused by vascular obstruction and renal parenchymal damage induced by systemic hypertension; glomerular disease is considered exceptional [13]. Recent clinical studies [5, 14] support this contention. Hong et al. [5] found that 9.7% of TA patients with renal artery involvement developed chronic renal insufficiency over a 90-month follow-up period. Similarly, Obiagwu et al. [14] showed that renal artery revascularization procedures were effective in salvaging renal function in children with TA-induced renal artery stenosis. However, an autopsy study [15] on 25 TA patients showed that whereas non-specific, ischemic and/or hypertensive glomerular changes were present in 44% of kidney specimens, 56% showed specific glomerular pathologies, most commonly diffuse mesangial proliferative glomerulonephritis; extent of large arterial inflammatory infiltrates assessed by morphometric analysis was most in the latter condition suggesting a relationship between the two phenomena.

This report highlights some characteristic features of percutaneous interventions in TA. Firstly, the high rate of restenosis seen after bare metal renal artery stenting in TA. Secondly, repeat intervention, mostly balloon angioplasty alone, is usually successful in dealing with the problem of restenosis. Thirdly, stenotic lesions in TA may be very resistant to dilatation, but can be progressively opened up with serial balloon dilatations over multiple sittings. Lastly, spontaneous dissections and aneurysms seen in TA can be effectively treated by endovascular techniques with minimal morbidity.

## Conclusion

Renal-related percutaneous interventions can be effectively and safely performed guided by CO_2_ angiography in patients with TA and renal insufficiency. Such procedures result in relief of symptoms, better blood pressure control, improvement in left ventricular systolic function and recovery or stabilization of renal function. CO_2_ angiography is a useful adjunct to the interventional armamentarium available to treat patients with TA and renal insufficiency.
